# What are the mechanisms and contexts by which care groups achieve social and behavioural change in low- and middle-income countries? Group motivation findings from a realist synthesis

**DOI:** 10.1017/S1368980022001367

**Published:** 2022-10

**Authors:** Pieternella Pieterse, Aisling Walsh, Ellen Chirwa, Anne Matthews

**Affiliations:** 1 School of Nursing, Psychotherapy and Community Health, Dublin City University, Glasnevin Campus, Dublin, 9, Ireland; 2 Department of Public Health and Epidemiology, School of Population Health, Royal College of Surgeons in Ireland, Dublin, Ireland; 3 Faculty of Midwifery, Neonatal and Reproductive Health Studies, Kamuzu College of Health Sciences, Blantyre, Malawi

**Keywords:** Care groups, Community health workers, Peer-to-peer learning, Behaviour change, Motivation, LMIC, Realist research

## Abstract

**Objective:**

Infant and under-five mortality rates in low- and middle-income countries (LMIC) can be reduced by encouraging behaviours such as sleeping under insecticide-treated bed nets, exclusive breast-feeding for the first 6 months, regular handwashing, etc. Community-based volunteer or peer-to-peer mechanisms are cost-effective ways of promoting these lifesaving practices. However, the sustainability and reach of community-based behaviour change promotion remains a challenge. Our inquiry focuses on the utilisation, by non-governmental organisations (NGO), of Care Groups, a peer-to-peer behaviour change intervention. We asked: What are the mechanisms and contexts by which Care Groups achieve social and behavioural change in nutrition, health and other sectors?

**Design:**

Realist synthesis reviewing forty-two texts that contained empirical evidence about Care Group interventions.

**Setting:**

LMIC.

**Participants:**

We held consultations with a research reference group, which included Care Group and nutrition experts, and Care Group – implementing NGO staff in Malawi.

**Results:**

Different types of motivation drive the establishment and the sustainability of peer group interventions. A certain amount of motivation was derived from the resources provided by the NGO establishing the Care Groups. Subsequently, both volunteers and neighbourhood group members were motivated by the group dynamics and mutual support, as well as support from the wider community. Finally, volunteers and group members alike became self-motivated by their experience of being involved in group activities.

**Conclusions:**

When designing and implementing community-based behaviour change interventions, awareness of the multi-directional nature of the motivating drivers that are experienced by peer- or community group members is important, to optimise these groups’ reach and sustainability.

Many low and middle-income countries (LMIC) have experienced significant improvements in under-five and infant mortality in the past two decades: The number of child and adolescent deaths (of all causes) decreased by 51·7 per cent between 1990 and 2017^(1)^. The greatest reductions in child mortality in LMIC have been related to a drop in respiratory infections, enteric infections (viruses, bacteria and parasites that cause intestinal illness), malaria, neglected tropical diseases and nutritional deficiencies^(2)^. Improvements in curative healthcare in LMIC have been accompanied by a focus on the prevention of diseases, through vaccines, preventative medicine and behaviour change communication: The promotion of, for example, good infant nutrition practices, ante- and post-natal care and improved household hygiene and sanitation are now a widely accepted components of public health^(3)^. Behaviour change interventions can be defined as ‘coordinated sets of activities designed to change specified behaviour patterns. In general, these behaviour patterns are measured in terms of the prevalence or incidence of particular behaviours in specified populations’^(4)^. Successful behaviour change interventions have promoted greater use of mosquito nets, increased facility-based deliveries, exclusive breast-feeding of babies for their first 6 months, and frequent handwashing with water and soap, which have reduced mortality related to malaria, childbirth, malnutrition and diarrhoea^(5–7)^.

While many potentially life-saving changes in behaviour can be accomplished at no cost or at a very low cost, bringing about lasting changes in behaviour remains a challenge. The delivery of behaviour change messages is labour intensive and can therefore be costly in terms of the deployment of personnel. To ensure that behaviour change messages reach the relevant individuals, LMIC governments (and non-governmental organisations (NGO) that engage in behaviour change communication) often use volunteer health promotors to pass them on^(5)^. Volunteer health workers, often called community health workers, have been shown to successfully deliver behaviour change communication when they are provided with training, small incentives and/or supportive supervision^(8)^. Community-based, peer-to-peer or volunteer-led family support programmes such as mothers’ groups, positive deviance/Hearth groups can effectively provide ante-natal care promotion, breast-feeding, hygiene and infant nutrition support^(5,9)^. There are some examples where this has worked well at scale, such as the Health Extension Worker program in Ethiopia^(10)^. Despite the positive impact of behaviour change support in terms of mortality and morbidity among young children, few community-based interventions are sustained in a cost-effective manner and adopted as part of the routine public health support package^(11)^ for pregnant women and mothers with children under five. As a result, community-level volunteer-led health interventions struggle with the issue of sustainability of the volunteer effort over time^(12)^. There are different reasons for this, from a lack of political will, no finances to scale up, to ill-fitting health policies and unsuccessful pilot programmes^(13)^. There is a surprising lack of in-depth studies that provide guidance to programme designers or could be used to create evidence-based policies^(14)^. Little research has been conducted into community motivation as a factor that can significantly influence the sustainability of volunteers^(15)^.

Our study of the Care Group approach, a volunteer-led, community-based, peer-to-peer model used primarily for health promotion, provides a close examination of the drivers of both volunteer and community-based participant behaviours within peer-to-peer learning interventions. We believe that the outcomes provide important lessons for the design of community-based volunteer health interventions, by providing insights into how motivation can be cultivated and sustained.

The Care Group approach was developed as an intervention to promote peer learning among neighbouring mothers by sharing knowledge and practice on exclusive breast-feeding, weaning and household hygiene^(16)^. The Care Group approach is primarily implemented by international NGOs, which utilise a cascading model whereby community-based mothers are elected by their peers to become Care Group Volunteers (CGV). The CGV receive monthly lessons within a group (the so-called Care Group), and each CGV is expected to pass this knowledge on to a group of approximately 10–12 other women in her neighbourhood (who form the ‘Neighbourhood Group’). The NGO usually recruits paid ‘promotors’ to teach CGV a new monthly lesson to pass on and carry out regular supportive supervision. This ‘cascading model’ uses a relatively small number of paid employees (promotors) to reach a significant number of women with children under two on a monthly basis (see Fig. 1).

**Fig. 1:**
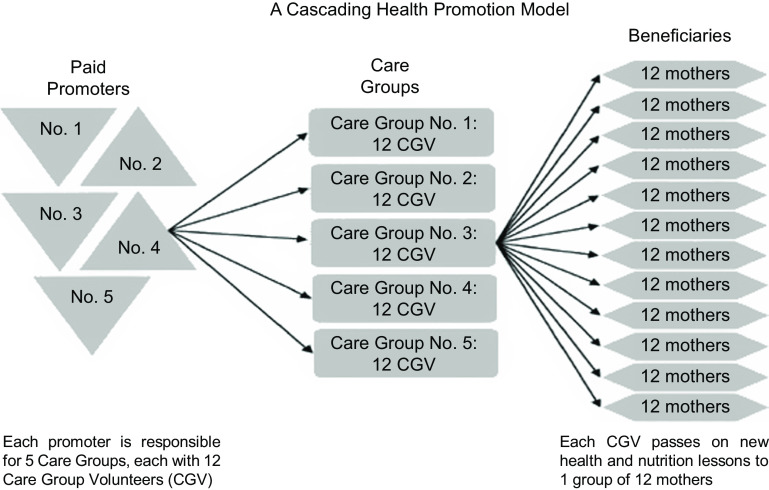
The Care Group model (© Davis *et al*., 2013)

The Care Group approach was developed by the NGO World Relief in 1996 in post-war Mozambique and has since been applied in over thirty countries^(16)^. Evidence suggests that Care Group interventions have been highly effective in a range of different settings^(17,18)^. The focus of many Care Groups was initially basic infant nutrition and primary healthcare, but in recent years, Care Group curricula are known to have included gender-based violence, post-natal depression, child protection and most recently COVID-19 prevention^(19,20)^.

We conducted a realist synthesis of international literature pertaining to Care Groups to better understand how Care Groups work and how they achieve their results. A realist synthesis is a theory-driven, interpretive approach to the review of evidence. It aims to produce explanatory analyses, that is, about what works, for whom, in what circumstances, and in what respects^(21)^. This article presents the findings of a realist synthesis of the literature on Care Group programmes. Realist research is an approach that allows researchers to establish which mechanisms led to the outcomes that were contributed to by certain interventions, in certain contexts^(22)^. This paper aims to answer the first question contained in our research protocol: ‘What are the mechanisms and contexts by which Care Groups achieve social and behavioural change?’^(23)^. This study fills a knowledge gap in relation to understanding of why, how and under what circumstances Care Group programmes achieve their outcomes. It provides insights into how community-based volunteers can be motivated and how this motivation can be sustained over time.

## Methods

The realist synthesis we conducted followed the steps set out in our research protocol^(23)^. These, are comprised of (i) developing our initial programme theories: a series of statements that describe components of the intervention under study and how we hypothesise that each will bring about change. We started out with a small number of initial programme theories but added additional programme theories during the data extraction phase and (ii) a literature search: A librarian assisted us with database searches across a range of bibliographic databases of most relevance to health, behaviour change and nutrition including Medline and PsychInfo. Sample search strategies and sample results can be found in supplemental file 1. As we described in the protocol for this synthesis^(23)^, there were challenges with the search as the terms ‘Care’ and ‘Groups’, and even ‘Care Groups’ are used in many different ways and contexts. As can be seen in the sample searches included, we specifically searched for articles using keyterms for the intervention (Care Group and variations), the focus of the intervention (nutrition, behaviour change, infant feeding, etc.) and also the geographical contexts. The key domains of interest did not map well on to any existing controlled terms of subject headings (especially ‘Care Groups’). Equally, related terms, such as community volunteers, were too broad in scope. We tried several approaches, including limiting our key terms to the title, given the likelihood that articles about Care Groups would be likely to indicate this.

The literature searches also included a search for ‘grey literature’: We consulted a website where Care Group intervention practitioners share knowledge. The majority of known Care Group publications could be found via this website, mostly in the form of evaluation reports and guidance documents^(24)^. In addition, we searched the database of the nutrition-focused publication Field Exchange, which is produced by the Emergency Nutrition Network and targets professional practitioners of nutrition-focused development aid interventions in LMIC (of which the Care Group approach is one type)^(25)^. Finally, we established a reference panel for our realist synthesis, which included two experts in the field of Care Group interventions, who further suggested titles to include. We hand searched the references of all Care Group publications to check for titles we had missed; (iii) screening of literature using inclusion and exclusion criteria. Included texts were those with: (a) some focus on and findings related to the implementation of a Care Group approach; with (b) concrete methods and/or the implementation processes, as practiced in the field, have been described – with references to when and where; and with (c) findings are presented from Care Group fieldwork; Excluded were those texts: (d) without empirical evidence, texts that provide only general guidance on how to set up/implement Care Groups; and (e) texts that make general statements about what Care Groups have achieved without these claims being linked to a particular intervention (not enough detail, no references to actual places and dates); (iv) data extraction. Four researchers (PP, AW, AM and EC) worked in pairs, iteratively coding all forty-two texts in Excel (each text was coded twice) by extracting relevant sections of the text and placing them under a series of IPT headings, which we added to every time additional theories or themes seemed to emerge. (v) data analysis and synthesis. Several readings of all extracted codes led to the confirmation of some initial programme theories and the merging and addition of new theories (we therefore drop the term ‘initial’ as our final set of theories are a mix of initial programme theories and those we added at the data analysis/synthesis phase). We used inductive, deductive and retroductive reasoning to produce all candidate theories^(26)^. Consensus emerged after several team discussions. Programme theories are underpinned by one, or a series, of statements that further dissect what is likely to happen when an intervention is introduced into a context (C) and creates or sparks a mechanism (M) that causes an outcome (O). These statements are usually referred to as CMO-configurations, or CMOC^(27)^. After agreeing on the programme theories, all relevant codes underpinning each CMO configuration were moved to individual Microsoft Word documents, to clearly demonstrate the evidence for our theories. We then held a feedback session with our practitioner reference group to ‘test’ our theories on the Care Group practitioners to see if they resonated. We made text adjustments to our programme theories where needed. (vi) write-up. We were guided by the RAMESES publication standards^(28)^ to draft this and other research publications.

## Results

**Fig. 2:**
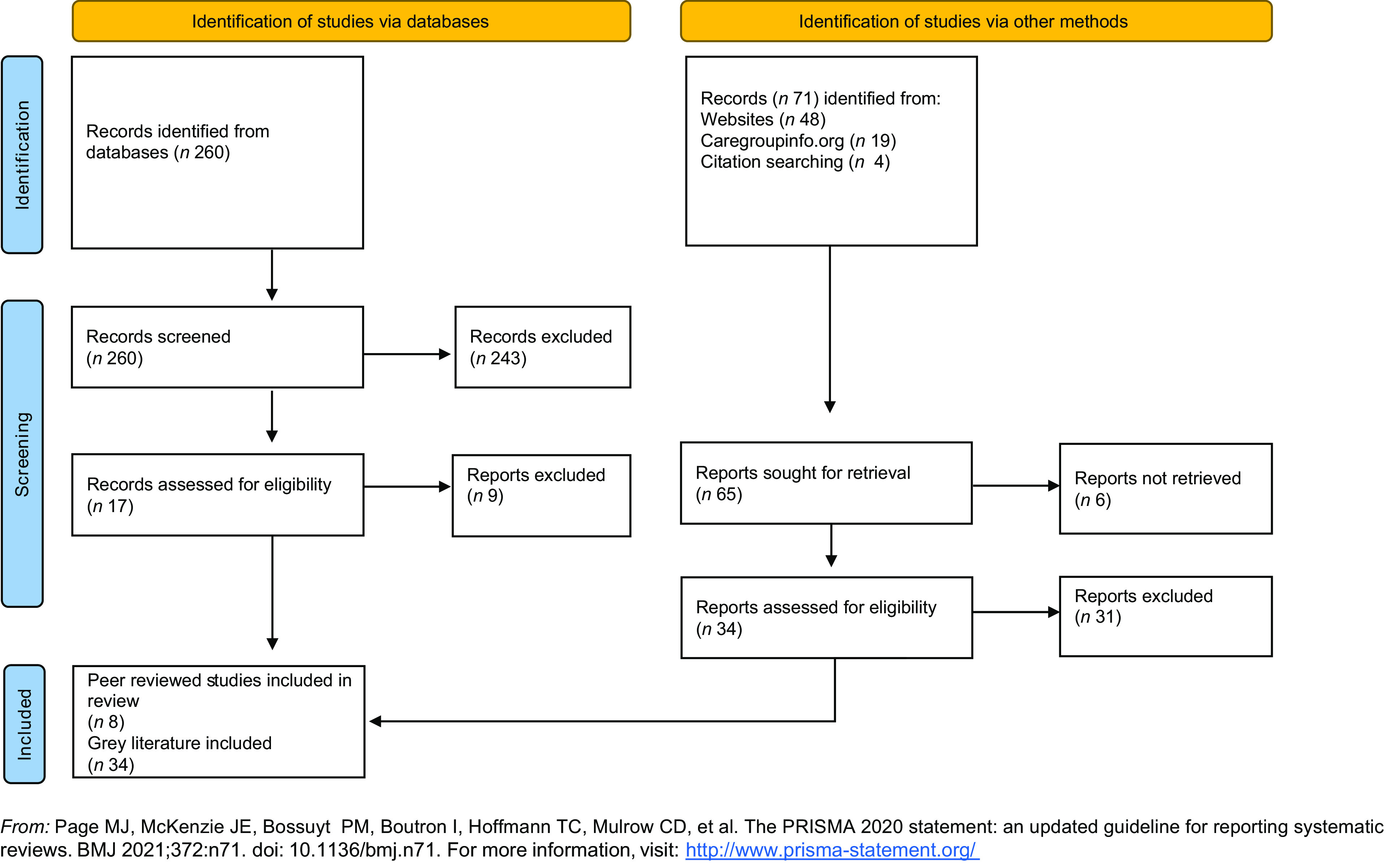
Flow chart of the literature search

The flow chart (Fig. 2) shows the total number of papers included at title and abstract screening stage via all database searches, and once duplicates had been removed (*n* 260). On full-text review, only seventeen of those were included. It also shows the results of our grey literature searches, which added a greater number of relevant texts.

From the forty-two articles included in the review, eight were peer reviewed articles and thirty-four ‘grey literature’ pieces: twenty-two programme evaluations, ten case studies and two documents outlining ‘how to implement Care Group interventions’ that included empirical data (see supplemental file 2 for the bibliography of included texts).

### Programme theories and context-mechanism-outcome configurations

Realist research works with (initial) programme theories. The objective of realist research is to prove, disprove or refine these theories. Among our aims is providing individuals who design Care Group programmes insights into what components could be adapted to improve these interventions. By focusing on the ‘how, why, when, for whom and to what extent’ an intervention works, realist research often leads to the emergence of much needed contextual nuance in reviews of interventions^(29)^. We developed nine programme theories and constructed eleven CMOC (see Table 1). In total, six out of the eleven CMOC related to volunteer motivation, which we focus on in this paper, as these findings add new insights to our understanding of Care Groups, and may be generalisable to other community-based volunteer interventions. The remaining theories related to the design and resourcing of Care Groups and the importance of a conducive environment for behaviour change messages to be accepted, these theories will be explored in a separate publication.

**Table 1:** The nine initial programme theories and eleven context-mechanism-outcome configurations

(Initial) program theories (IPT) and context-mechanism-outcome (CMO) configurations for the Care Group realist synthesis			
CMO	Context	Mechanism	Outcomes
	*Care Group Volunteer*		
IPT-1:	If a CG programme provides Care Group Volunteers (CGV) with a manageable workload and incentives (financial or non-financial), then CGV can have higher levels of self-efficacy and sense of empowerment as a result of their engagement with CG Neighbourhood Group Members (CG-NGM), pride in their work and motivation, which can lead to low attrition rates.		
CMO-01	Manageable workload, time to build relationships, T-shirt, umbrella, flipcharts, possibly savings and loans groups	Motivation (external, provided by project)	Low attrition rate among CGV
CMO-02	Regular supportive meetings with other CGV, regular supportive supervision, being elected to CGV by peers, community (+leadership) praise, recognition	Positive peer pressure, external validation, provided by community	Stronger relationship w community group members Low attrition rate among CGV
CMO-03	CGV training, CG experience, regular supportive supervision, positive feedback due to health improvements, interaction w healthcare staff	Confidence, self-efficacy	- Low attrition rate among CGV - (Sometimes) shift in gender dynamics in household
	*CG neighbourhood group members*		
IPT-2:	In a context where little health-related information is available, if a local woman who lives nearby gets elected CGV, receives training and is willing and able to provide regular, accessible ‘healthcare’ lessons, then her neighbours (CG-NGM) are likely to attend CG-NGM meetings, be open to advice, consider the messages, with increased likelihood of behaviour change.		
CMO-04	Local/familiar/same ethnic or religious group CGV, acceptance of message, accessibility of messages, regular repetition of core messages	Trust, understanding, accessibility and acceptance [of health messengers]	CG-NGM attend meetings, hear the messages and consider behaviour change
IPT-3:	In contexts where women face similar challenges to nutrition and breast-feeding and where a large proportion of eligible women attend the CG-NGM meetings, the group-based learning and sharing of positive/successful examples of nutrition or breast-feeding can enable other participants to follow their examples and adopt positive health strategies.		
CMO-05	All women who are pregnant or have children under 2 (*5 years old in some locations) are invited to attend CG-NGM meetings, and most women within communities attend meetings, trying or adopting new behaviours is encouraged by peer group, positive results of behaviour change are experienced or witnessed in others	Positive peer pressure	Trying of new behaviours, adoption of new behaviours, encouraging others to try new behaviours
IPT-4:	In a context where CG-NGM regularly attend CG-NGM meetings to learn about improved health behaviours, to share their stories, problems, then they experience a sense of empowerment from this social contact, are likely to continue to attend such meetings, adopt healthy behaviours, continue to feel empowered and may experience a shift in gender dynamics within household.		
CMO-06	Regular CG-NGM meetings well attended, social bonds between CG-NGM established, possibly additional (cooking, loans/saving) activities engaged in	Empowerment, social cohesion	Sustainability of group structure, empowerment + self-respect, increased community/group cohesion
	*Extended family, village leadership*		
IPT-5:	If in a context where husbands, mothers (-in law) and local traditions have huge influence over women’s reproductive and child rearing decisions, Care Groups Volunteers engage and gain the trust of the community and the community leadership, then the CG-NGM experience more freedom to adopt new, healthy behaviours, even if they deviate from traditional practice.		
CMO-07	Husbands, mothers-in-law, elders and others in community have influence in a woman’s decision making regarding reproductive health. In this setting, Care Group Volunteers create relationship with these ‘gate keepers’ and influencers, engage them in their lessons, demonstrate effectiveness of behaviour change.	Acceptance, empathy (of ‘power holders’, e.g. husbands, mothers (in-law))	- Women allowed to, encouraged to adopt healthy reproductive, maternal and child health (RMCH) behaviours, modern medicine. - Reduction in traditional beliefs regarding sickness being, e.g. due to spells cast by others or by women’s promiscuous behaviour.
	*Health facility, community case management providers*		
IPT-6:	In contexts where CGV act as linkages between community and health facilities or other health system structures such as community health workers then more connections and communication can occur promoting cohesion across services leading to higher coverage and great uptake of interventions.		
CMO-08	CGV are linked with local health facilities and/or CHW, formal or informal networks are created, CGV link back and forth between community, CG-NGM and other care providers, passing on vaccination schedules, collect vital events data, create confidence in health services, bring health service providers to communities by facilitating outreach visits.	Facilitating connections, promoting confidence in Ministry of Health (MoH) healthcare delivery	- Greater uptake of MoH services - Sustainability of Care Groups if integrated/linked to existing (paid) MoH staff such as CHW; sustainability of improved healthcare uptake if citizens experience MoH health services for first/sustained time
	*Ministry of Health, District/Provincial Health Authorities*		
IPT-7:	In contexts where MoH are involved in supporting CG structures, this can lead to buy-in for the CG intervention, supporting a more sustainable CG structure.		
CMO-09	MoH at national or sub-national level are actively engaged in supporting Care Group structures, have nominated individuals to take responsibilities for teaching and supportive supervision tasks.	Facilitation and/or formal integration of new responsibilities	- Training, support and supervision of CG taken on by MoH staff, CG structure and work sustained. - Establishment of community-based drug distributors or similar bringing increased primary healthcare demand and supply in balance.
	*NGO, NGO staff, CG programme design*		
IPT-8:	If, in a Care Group programme context, the implementing NGO carefully designs and funds the intervention, with sufficient staff to provide supportive supervision to promotors and CGV, then CGV are likely to motivated and confident that can lead to them creating a conducive environment for achieving behaviour change.		
CMO-10	Well-designed CG programme. Sufficient funding for supervisory staff to visit field staff routinely, sit in on CG, checking on quality of messages CGV pass on, funding for staff training, refresher training, etc.	CGV confidence due to conducive, supportive environment	CGV successfully pass CG messages, inspire confidence in their conviction to change behaviours.
IPT-9:	If, in a context where local communities may use harmful traditional practices, have little knowledge about how common illnesses are caused the implementing NGO conducts behavioural surveys before the curriculum is designed and implemented and/or collects vital events data and uses this to feedback to community and adjust the programme if needed then these Care Groups have a high chance of being successful in targeting behaviour changes that are most urgently needed, hereby promoting access to modern healthcare and reducing reliance on traditional (healer) practices.		
CMO-11	Community that uses harmful traditional practices, believes in witchcraft-type health superstitions, lacks sufficient healthcare information and understanding. Care Group intervention is based on formative research (e.g. baseline survey) and behavioural lessons are targeted according to survey outcomes. Gathering of vital events data and share these with community to provide evidence that behaviours they promote improve community/infants’ health.	Precise targeting and persuasion of community, backed up by understanding of community’s traditional practice and evidence of health improvement	- Reduction in traditional practices, reduction in reliance on traditional healers, no blaming of community members for casting spells or blaming women’s promiscuous behaviour for children’s ill health. - Greater understanding of causes of illness, greater adoption of healthy RMNCH behaviours.

ITP = initial programme theory; CMO = context–mechanism–outcome; CGV = care group volunteer; CG-NGM = care group neighbourhood group member.

The objective of Care Group interventions is the peer-to-peer transfer of knowledge and behaviour change messages from the CGV to her neighbourhood group, with the ultimate goal being behaviour change adoption by the neighbourhood group members (CG-NGM). All Care Group texts we included in our study were successful in achieving some, or all, of their project aims, which were measurable behaviour changes in the project target population. The forty-two texts included in our realist synthesis were largely focused on the CGV and the CG-NGM, which are at the heart of all Care Group interventions. The design of the large majority of Care Group interventions focused on establishing a system of volunteers who are committed to their groups and receive only some small material incentives for their efforts. Second, they focused on how to keep CGV motivated and on-task month after month. From the implementing NGO’s perspective, the CG-NGMs’ attendance at group meetings is the second most crucial element of a successful programme. Care Groups and neighbourhood groups are established in the first instance by (paid) NGO staff who act as so-called ‘promotors’ (direct supervisors of CGV and trainers of their monthly lessons), who are usually from the area where the intervention takes place and who are crucial for supportive supervision. Context-specific questions arise for every intervention; how to best provide training for CGV, what types of small incentives (such as a T-shirt identifying CGV, umbrellas, bags) and flip charts as teaching aids, are selected, and what curriculum is chosen^(30)^. Ensuring that ‘supportive supervision’ is culturally sensitive and carried out regularly is crucial to ensure continued motivation^(31)^. Evidence suggests that well designed and well executed Care Group interventions can lead to a range of significant behaviour change outcomes^(17,18)^. Selected Care Group texts included reviews and case studies of interventions from over a dozen different locations, from Cambodia to Guatemala, Senegal and Malawi. During the coding phase of the research, clear patterns of common challenges and opportunities started to emerge, no matter where the interventions were conducted.

The realist research approach allowed us to examine the various CMO configurations that are observable in relation to CGV. We divided the entire body of evidence regarding the CGV into three different CMO configurations, whereby the mechanism (M) was the distinctive feature and the outcome (O) remained more or less constant throughout, while the context (C) varied to some extent. Each CMO configuration is in fact a theory that explains what researchers believe has happened to create outcome O, which was the result of mechanism M having been activated by an intervention that was introduced to context C.

### Motivation in all its nuances

What emerged in relation to Care Groups was a more complex picture than was apparent within the existing evaluations, as three distinct and observable sources of motivation and empowerment were revealed in relation to CGV. Moreover, we saw this mirrored in relation to the CG-NGM. CMO-01 shows that in a context (C) where women who are elected CGV and are provided with a group meeting structure, a manageable workload, a T-shirt or wrap that distinguishes them from others in her group, flipcharts and a bag to carry their Care Group items (which are all provided by the implementing NGO), these women can be motivated by this, which will encourage them to remain engaged in the program. One of the articles noted ‘the training they received, the positive changes in community health behaviours, and the recognition they received for their contributions to their communities were the factors that motivated them to continue their work. They reported that they felt that their workload as volunteers was manageable… The volunteer dropout rate has been very low in spite of very limited material support from the project’^(32)^.

In addition, we repeatedly found that recognition from senior members within the community and positive feedback from grateful neighbourhood group members lead to CGV experiencing positive peer pressure from everybody involved in the Care Groups and neighbourhood groups: ‘They [CGV] were also motivated to see the health of people in the community improve. They loved to be recognized as health workers in the community, though at times they felt like they were being considered like doctors’^(33)^. CMO-02 therefore focuses on the motivational effect that is derived from praise, recognition by peers, community and community leadership. This is especially so when CGV regularly meet the other CGV during Care Group meetings and in a context whereby CGV receive supportive supervision. In addition, external validation provided by community members and community leadership often leads to CGV feeling respected by the community generating motivation to continue acting as a volunteer^(34)^.

CMO-01 describes how the CGV motivation can originate from the NGO, while CMO-02 describes the CGV’s motivation coming from community members. CMO-03 shows that in the context where women who have become CGV have received training, hold meetings with staff from the local health facility, regularly meet with their neighbourhood groups, see positive changes and experience a good atmosphere among the women in their groups, these women feel confident and gain a sense of self efficacy, which motivates them to remain active within their Care Groups^(35,36)^. In a Care Group intervention in Cambodia, the volunteers were involved in the development of a new breast-feeding strategy for the Cambodian Red Cross (the implementing agency) which elevated the status of many of the women involved ‘[the volunteers], along with other key women in the village, to take on more responsibility for training volunteers in their village. This empowering approach increased teamwork as well as capacity and status for the key women within their communities’^(37)^.

This increased self-confidence among CGV seems to regularly create a positive shift in gender dynamics in the household. Research conducted by a Care Group implementing NGO showed that ‘the proportion of CGV who had accepting attitudes towards domestic abuse was only 3 % at the end of the project compared with 24 % of the beneficiary mothers whom they served’ (p. 44)^(20)^.

As Table 1 shows, the similarities between the three CMOC constructed for CGV and the three for the CG-NGM are striking. CMOs-04, -05 and -06 logically enough culminate in the same overall outcome (O), which is the continued attendance of the CG-NGM meetings. The contexts (C) across CMOs-04, -05 and -06 are similar too. Like CMO-01, -02 and 03, the mechanisms (M) are all slightly different depending on who ‘drives it’, and across both groups (CGV and CG-NGM) we see motivation to attend neighbourhood groups being derived from the right NGO’s incentives (CMO-01 and CMO-04), from peer pressure (CMO-02 and CMO-05), and over time, we see the emergence of self-efficacy and empowerment generated from within the neighbourhood groups (CMO-03 and CMO-06).

For CG-NGM, CMO-04 represents a context in which women in mainly rural areas have little or no access to health information. In this context (C) an NGO establishes a Care Group program that invites local women to nominate CGV, giving the women say in who will represent them. The chosen volunteers are naturally familiar to the community and receive health information to pass on that is accessible and easy to understand. For women who are not literate, the solutions that are presented are communicated via flip charts with pictures. Importantly, the solutions provided are achievable, e.g. weaning a 6-month-old baby with locally available supplementary foods. The mechanisms (M) are trust, understanding and acceptance of the messengers and the messages, which lead to outcome (O) of continued attendance of CG-NGM meetings and attempts at trying out the suggestions discussed during the group meetings: ‘An overwhelming majority of both leader and beneficiary mothers reported that they would be able to implement at least some of the lessons that they had learned at their care group meetings… Hygiene, which includes washing hands using a tippy tap [a low-cost handwashing contraption made with a plastic water bottle]…was the lesson most often mentioned as being implementable by both leader and beneficiary mothers’^(38)^. The Care Group texts highlighted the importance of working with trusted local individuals who inform women and can gain the trust of household decision makers such as husbands: ‘Understanding the lessons and reviewing lessons at their care group meetings, having the necessary materials and having support from their husbands were the things that were reported as being most likely to help beneficiary and leader mothers implement the lessons that they learned during their care group meetings’^(38)^.

For CMO-05, the context and outcome were similar to CMO-02, which focuses on the peer group element of the neighbourhood groups, emphasising camaraderie and positive peer pressure felt when all women with children between the age of 0 and 2 years, simultaneously try to adopt the new behaviours. One programme report noted that ‘As more community members accept and begin to practice [behaviour change] and discern the benefits, more pressure tends to mount on those who have not embraced the [behavior change] to comply… For instance less and less people in the project area seek treatment for fever and convulsions from traditional healers now than before the project and much less people reveal they visited a traditional healer for fear of stigma and ridicule that now comes with such care-seeking behavior’^(39)^. The texts revealed a number of instances whereby Care Group lessons promoted practices that are beneficial to children but contravened local tradition, allowing women to adopt ‘…good practices that communities previously deemed unacceptable… pregnant mothers now “openly and defiantly” continue breast-feeding their children and much more pregnant mothers and children eat eggs’^(39)^. In a Care Group intervention in Senegal, it was noted that the group members started to resist certain community norms and gender dynamics^(40)^.

Finally, CMO-06, like CMO-03, shows that empowerment and social cohesion are the mechanism(s) (M) that are generated as a result of many neighbourhood group activities, which lead to the outcome (O) of sustainability of group structure, self-respect and increased community/group cohesion: ‘People are now taking increased responsibility for each other, and they have more compassion collectively for the poor. Because of the project, mothers now feel that they are now a more important part of the community’ (p. 117)^(41)^.

In realist syntheses, researchers ‘test’ CMOC by identifying evidence in the texts that confirms the theory (CMOC) applies. At times, it is also possible to test a CMOC by showing that the absence of a certain element within a context leads to a lack of outcome, or use a combination of both approaches, which is called deductive and retroductive reasoning^(26)^. For example, to confirm CMO-01 and CMO-04 (the theories linking the support provided by the Care Group NGO and the success in achieving group motivation and eventual behaviour change outcomes), we identified several examples, whereby a lack of support for the Care Groups was linked to their eventual failure to achieve the desired behaviour change outcome at scale: In one project review, the authors of the report noted that the implementing NGO should ‘…focus more on the quality of Care Group activities, recognizing that messaging is not enough to lead to sustained behavior change. Greater attention is needed to improve counseling and negotiation for mothers who are experiencing problems with child feeding and caregiving’^(42)^. Evidence suggested that implementing NGO sometimes focused too much on training the volunteers but not enough on quality checks to see how well CGV conducted group and house visits in the community: ‘Much greater attention is still needed to train the volunteers in good nutrition counseling skills and on how to make home visits effective, starting with visiting the homes of the right age group’^(43)^.

## Discussion

In realist research, ‘formal theory is often used to identify mechanisms and features of context and to explain how overall (often apparently disparate) sets of findings fit together’^(44)^. When motivation emerged as a theme throughout this research, we then reverted to existing theories of motivation to verify that the findings we extracted and conclusions we described in our CMOC were plausible. We noted significant similarities between CMOCs 01-06 and Frederick Herzberg *et al.*’s motivation-hygiene theory^(45)^. It is clear that what motivates CGV and CG-NGM initially are a range of support measures that allow both sets of participants to have the skills and capabilities to attend, combined with a manageable workload/meeting schedule. This is designed to ‘not discourage’ CGV from fulfilling their monthly responsibilities and for CG-NGM to attend local meetings. This is similar to what Herzberg and colleagues termed ‘hygiene factors’. Their research into what motivates people to work hard and to produce their best efforts concluded that there are a certain number of factors (so-called hygiene factors) that have to meet a minimum standard in order to ensure that certain work (including voluntary) can be done^(40)^. A failure to meet these minimum standards (by, for example, a heavy workload) creates job dissatisfaction. However, when these minimum standards are met, they rarely create satisfaction or motivation; they simply make it possible to complete the job.

Herzberg *et al.* discovered that the things that motivate people to work are factors of a different magnitude, things that speak to a different emotion within people; self-actualisation, recognition, learning new things or achieving ambitions^(45)^. In Care Group settings, we see similar patterns, CGV and CG-NGM who host or attend what appear to be successful groups are recorded as noticing a sense of empowerment within the group, recognition by family, village leadership and health workers. These are clearly the factors that motivate them to continue attending Care Group meetings and ultimately create a sustainable peer-supporting environment.

Considering CMO-01 and CMO-04, in both cases, the implementing NGO provides CGV and CG-NGM with a series of incentives, training and opportunities that allow Care Groups to become established. The basic monthly lessons, the small incentives, the manageable workload and the culturally and locally acceptable health messages delivered by trustworthy local sources are all hygiene factors that make it possible and interesting for all those involved to continue as volunteer or to attend regular meetings. However, in most cases, it is hard to imagine that increasing the intensity or frequency of these factors would lead to greater motivation. CMO-02, 03, 05 and 06, on the other hand, deal with supportive supervision, interaction with health facility workers for CGV, peer group validation, and the actual group project of adopting new practices for group members, which lead to a greater sense of empowerment and self-efficacy, both among volunteers and neighbourhood group members. Such motivation is reflected in the Care Group literature that speaks of a person’s life having ‘changed because I feel respected in the community’^(41)^, and of empowering approaches that ‘increased teamwork as well as capacity and status for the key women within their communities’^(37)^.

The overlap between the CMO constructed for our realist synthesis and Herzberg *et al.*’s motivation and hygiene theory strengthens the conclusion that three types of motivational drivers are important for the achievement of successful Care Group programmes, namely: (1) NGO provide the basic training and logistical support for the establishment of Care Groups. They also implement a suitable design that takes account of workload, distances between community members and well-chosen incentives for CGV. This support needs to be well-structured, but this alone may not sustain motivation over time; (2) Peer and social pressure drivers of motivation seem most effective in Care Group programmes that reach the large majority of the women of reproductive age within a community – the so-called census approach^(46)^. Ensuring that a critical number of women meet regularly is crucial for this type of motivation to be generated from within the group, while the wider community needs to be made aware of the CG achievements, which should generate appreciation and praise for the volunteers. Finally (3), internal motivation, empowerment and a sense of self efficacy are generated by the regular contact between CGV and CG-NGM, which is facilitated by the training and supportive supervision they have received. These sources of empowerment and self-efficacy are further strengthened by evident improvements in the communities’ children’s health and reinforced by the respect received from other community members, including from their husbands or mothers-in-law.

Insights into the factors that might influence the motivation of CGV and CG-NGM should guide the way NGO that implement Care Groups support these structures. The examples of programme reviews where Care Group interventions did not achieve the results that an NGO had hoped, showed evidence of a lack of awareness of the motivation and group dynamics. Care Groups rely on supportive supervision, check-ins at all levels to establish if the neighborhood groups are meeting, what the groups are discussing and the health measurement data they are collecting. These are programme instruments, but they can also be sources of motivation that inspire CGV to keep organising group meetings.

## Conclusion

Care Groups are a useful approach to peer-to-peer learning that can play an important role in the promotion of lifesaving health, hygiene and nutrition behaviours in LMIC. Our realist synthesis of Care Group literature has brought to light the intricacies of group motivation and how important the various drivers of motivation may be for the success and sustainability of what can be a low-cost and self-sustaining community-based initiative. The similarities between Herzberg *et al.*’s motivation and hygiene theory and the CMOC tested and verified by this research show that most individuals respond to motivation from their environment, their peers and ultimately their own self-esteem, regardless of whether a person is in a factory in Pittsburgh or in a rural community in Mozambique or Cambodia.

Whilst programme design is only one part of a much larger puzzle faced by LMIC governments that aim to scale up community-based health volunteer structures, using the Care Group model as a blueprint for a Ministry of Health-embedded community volunteer programme^(47)^ has demonstrated that scaling the Care Group approach is possible, as long as the drivers of volunteer and participant motivation can be kept in check.

## Supplementary material

For supplementary material accompanying this paper visit https://doi.org/10.1017/S1368980022001367. click here to view supplementary material
