# Supplementary material for: What are the mechanisms and contexts by which care groups achieve social and behavioural change in low- and middle-income countries? Group motivation findings from a realist synthesis
Source: Public Health Nutr. 2022 Jun 1;25(10):2908–19. doi: 10.1017/S1368980022001367 (PMC9991844; doi:10.1017/S1368980022001367)
Supplement: Supplementary file 1 [file S1368980022001367sup.zip › S1368980022001367sup001.docx]

**Motivation in Care Groups- Supplementary file 2: Bibliography of articles and documents selected for inclusion in the review**

- 1. Adler, A. (2017) ‘From beneficiary to agent of change, Save the Children Malawi’, pp. 6–7. Available at: <https://malawi.savethechildren.net/news/beneficiary-agent-change>.
  2. American Red Cross and VSO (2007a) *Volunteer Participation and Mebendazole Consumption During an Integrated Measles Campaign: Siem Reap Cambodia. Integrated Child Health Project, Activity Report IV*.
  3. American Red Cross and VSO (2008) *Only the Breast: Development & Implementation of a Strategy to Promote Optimal Breastfeeding Behaviors in a Child Survival Project- Activity Report IX*.
  4. Beracochea, E. *et al.* (2007) *Curamericas/Guatemala Census-Based, Impact-Oriented Child Survival Project October 1, 2002 - September 30, 2007*. Available at: [file:///C:/Users/abezr/AppData/Local/Temp/2007 Curamericas Guatemala Final Eval.pdf](file:///C:/Users/abezr/AppData/Local/Temp/2007%20Curamericas%20Guatemala%20Final%20Eval.pdf).
  5. Bessenecker, C. Kolesar, R. and Ram, S. (2007) *Final Evaluation Report: Integrated Child Health Project Siem Reap Province, Cambodia*.
  6. Best, R., Chassy, A., Chigwenembe, A., DeVries, M., Germain-Aubrey, C., Kieffer, M.P., Kumbukani Black, M., Manske, M., Mungoni, L., Mutiro, K., Sunseri, A. and Wilkerson, M. (2017) *Joint Mid-Term Review of the UBALE and Njira Projects; USAID-funded Title II Development Food Assistance Projects in Malawi led and Catholic Relief Services (UBALE) and Project Concern International (PCI).*
  7. Capps, J. M. (2006) *Rwanda “Umucyo” (Illumination) Child Survival Project Final Evaluation Report*.
  8. Capps, J.M., Carruth, M.H., Nitkin, T., Doty, D. and Dechasa, S. (2011) *Grand Cape Mount Child Survival Program Improved Child Health in a Transitional State through IMCI*, *Phys. Rev. E*.
  9. Capps, J. M. *et al.* (2009) *Plan International KIDCARE Child Survival Project, Kilifi, Kenya, Final Evaluation Report*.
  10. Casazza, A. L. and Hower, R. (2007) *Learning for Leverage : Scaling up from a CSP Final Evaluation with Lessons to Meet New Challenges in the Future Light for Life Cost Extension Child Survival Project*.
  11. CORE Group (2009) ‘Community Approaches to Child Health in Malawi—Applying the C-IMCI Framework’.
  12. Crespo, R., Kabaghe, V., Morrow, M. and Borger, S. (2009) *Tube Poka Child Survival Project Final Evaluation Report*, *Chart*. Available at: <http://www.caregroupinfo.org/docs/WRC_Malawi_CS_Final_Eval_2009.pdf>.
  13. Crigler, L., Gergen, J. and Perry, H. (2013) *Supervision of Community Health Workers*. Available at: <http://www.mchip.net/sites/default/files/mchipfiles/09_CHW_Supervision.pdf>
  14. Davis, A., Elmer, M., Ernst P., Hower, R., Morrow, M., Davis, T., Wetzel C., and Borger, S. (2010) ‘Key Document, 2010, Establishing Care Group Criteria’.
  15. Davis, T., Wetzel, C., Avilan, E.H. De Mendoza Lopes, C., Chase, R.P., Winch, P.J. and Perry, H. (2013) ‘Reducing child global undernutrition at scale in Sofala Province, Mozambique, using Care Group Volunteers to communicate health messages to mothers’, *Global Health Science and Practice*, 1(1), pp. 35–51. doi: 10.9745/GHSP-D-12-00045.
  16. Edward, A. Ernst, P., Taylor, C., Becker, S., Mazive, E. and Perry, H. (2007) ‘Examining the evidence of under-five mortality reduction in a community-based programme in Gaza, Mozambique’, *Transactions of the Royal Society of Tropical Medicine and Hygiene*, 101(8), pp. 814–822. doi: 10.1016/j.trstmh.2007.02.025.
  17. George, C.M., Vignola, E., Ricca, J., Davis, T., Perin, J., Tam, Y. and Perry, H. (2015) ‘Evaluation of the effectiveness of care groups in expanding population coverage of Key child survival interventions and reducing under-5 mortality: A comparative analysis using the lives saved tool (LiST) Global health’, *BMC Public Health*. BMC Public Health, 15(1), pp. 1–9. doi: 10.1186/s12889-015-2187-2.
  18. Haeften, R. Van, Caudill, H. and Kilmartin, E. (2013) *Second Food Aid and Food Security Assessment (FASFA-2)*. Available at: [www.fantaproject.org](http://www.fantaproject.org).
  19. Hillowis, H. (2016) ‘Improving nutrition behaviours through a Care Group Model project in Somalia’, *Nutrition Exchange, ENN*. Available at: nline.net/nex/6/improvingnutsomalia.
  20. Jennings, J. (2009) *GOAL ETHIOPIA Sidama Child Survival Program MID-TERM EVALUATION REPORT Awassa Zuria and Boricha Woredas of the SNNP Region of Ethiopia*. Available at: <http://www.caregroupinfo.org/docs/GOAL_CSP_ethiopia_MTE_report.pdf>.
  21. Kieffer, M. P. and Muula, A. (2019) *Final Project Report: Father Involvement in Promoting Reproductive, Maternal, Newborn, and Child Health (RMNCH)*. Available at: https://www.fsnnetwork.org/sites/default/files/Final Project Report - Father Involvement in Promoting RMNCH.pdf.
  22. Larkin, B. W. and Tanaka, J. (2018) ‘Training Care Groups on sexual and gender-based violence in rural Niger’, *Field Exchange - Emergency Nutrition Network ENN*, pp. 31–33. Available at: [www.ennonline.net/fex/58/trainingcaregroupsniger](http://www.ennonline.net/fex/58/trainingcaregroupsniger).
  23. Laughlin, M. (2010) *A Guide to Mobilizing Community-Based Volunteer Health Educators: The Care Group Difference*.
  24. MacDonald, M. E. and Diallo, G. S. (2019) ‘Socio-cultural contextual factors that contribute to the uptake of a mobile health intervention to enhance maternal health care in rural Senegal’, *Reproductive Health*. Reproductive Health, 16(1), pp. 1–12. doi: 10.1186/s12978-019-0800-z.
  25. Mokori, A. Hendriks, S.L., Oriskushaba, P. and Oelofse, A. (2013) ‘Changes in complementary feeding practices and nutrition status in returnee children aged 6-23 months in northern Uganda’, *South African Journal of Clinical Nutrition*, 26(4), pp. 201–211. doi: 10.1080/16070658.2013.11734473.
  26. Mutwiri, G. and Ndumi, A. (2016) *Implementing the care group approach: Tanzania case study*. London, UK.
  27. Ndungu, P. and Tanaka, J. (2017) ‘Using care groups in emergencies in South Sudan.’, *Field Exchange - Emergency Nutrition Network ENN*, (54), pp. 95–97. Available at: <http://files.ennonline.net/attachments/2592/FEX-54-Web_FEB-2017-SHARE.pdf>.
  28. Nyathi, C. (2019) *Care groups: providing health support, building confidence*. Available at: <https://www.unicef.org/zimbabwe/stories/care-groups-providing-health-support-building-confidence>.
  29. Olney, D., Parker, M.E., Iruhiriye, E., Leroy. J. and Ruel, M. (2013) ‘A Process Evaluation of the Tubaramure Program for Preventing Malnutrition in Children under 2 Approach (PM2A) in Burundi’.
  30. PCI (2016) *SPECIAL TOPIC: Care Group Trios in Bangladesh*.
  31. Perera, S. M. (2015) ‘Adolescent inclusion in the Care Group approach: the Nigeria experience’, *Field Exchange Emergency Nutrition Network ENN*, (July), pp. 110–112. Available at: <https://ovidsp.ovid.com/ovidweb.cgi?T=JS&CSC=Y&NEWS=N&PAGE=fulltext&D=caba6&AN=20163220205%0Ahttp://library.wur.nl/sfx_local?sid=OVID:cabadb&id=pmid:&id=doi:&issn=1743-5080&isbn=&volume=&issue=52&spage=110&pages=110-112&date=2016&title=Field+Exchange+-+Em>
  32. Perry, H., Ernst, P., Chitlhango, I., Machaila, A., Borger, S. and Morrow, M. (2009) *Expanded Impact Child Survival Program, final evaluation report, Gaza Province, Mozambique*. Available at: <http://dec.usaid.gov/index.cfm?p=search.getCitation&CFID=16930585&CFTOKEN=29828566&id=s_8D18334C-D566-FC5C-DB8C8F6F842DA957&rec_no=160886>.
  33. Perry, H., Summer, A., Boswell, C., Simutwe, P., Hamatanga, E., Dumbula, M., Kalinda, R. and Mwiinga, B. **(**2010a) *Chikankata Child Survival Project (CCSP), 2005‐2010 Final Evaluation Report*.
  34. Perry, H., Avilan, E.H., Lopes, C., Menete, L. Davis, T. and Wetzel, C. (2010b) *Expanded Impact Child Survival Program, Final Evaluation Report Sofala Province, Mozambique.*
  35. Rana, M., Huan, N. Van and Thach, N. N. (2017) *Effectiveness of community-based infant and young child (IYCF) support group model in reducing child undernutrition among ethnic minorities in Vietnam September 2017 List of Acronyms*.
  36. Robins, C. and Best, R. (2009) *I-LIFE Malawi Development Assistance Program*, *October*. Malawi. Available at: <http://www.caregroupinfo.org/blog/wp-content/uploads/2009/11/I-LIFE_Evaluation_28Jan09.pdf>.
  37. Sarriot, E. (2011) *Final Evaluation of the Kabeho Mwana Expanded Impact Child Survival Program*.
  38. Tura, H., Cornish D. and Roberts-Dobie, S. (2020) ‘Mother-to-mother: Evaluation of the sustainability of a peer-to-peer model to communicate nutrition messages in Mozambique’
  39. Weiss, J., Makonnen, R. and Sula, D. (2015) ‘Shifting management of a community volunteer system for improved child health outcomes: Results from an operations research study in Burundi’, *BMC Health Services Research*, 15(Suppl 1), pp. 1–9. doi: 10.1186/1472-6963-15-S1-S2.
  40. Wilner, L. Suri, D.J., Langlois, B.K., Walton, S.M. and Rogers, B.L. (2017) ‘Effective delivery of social and behavior change communication through a Care Group model in a supplementary feeding program’, *Journal of Health, Population and Nutrition*, 36(1), pp. 1–10. doi: 10.1186/s41043-017-0111-3.
  41. World Relief (2004) *Final Evaluation of the Tiweko Tose Child Survival Project, Malawi*.
  42. Yakowenko, E. and Pietzsch, S. (2017) ‘Learning from the Porridge Mums project in northeast Nigeria.’, *Field Exchange - Emergency Nutrition Network ENN*, (No.55), pp. 53–55. Available at: <http://www.ennonline.net/learningfromtheporridgemumsprojectinnortheastnigeria%0Ahttps://www.cabdirect.org/cabdirect/abstract/20173354314>
