# Supplementary material for: What are the mechanisms and contexts by which care groups achieve social and behavioural change in low- and middle-income countries? Group motivation findings from a realist synthesis
Source: Public Health Nutr. 2022 Jun 1;25(10):2908–19. doi: 10.1017/S1368980022001367 (PMC9991844; doi:10.1017/S1368980022001367)
Supplement: Supplementary file 1 [file S1368980022001367sup.zip › S1368980022001367sup002.docx]

**Motivation in Care Groups- Supplementary file 1: Sample search strategies**

Database(s): **Ovid MEDLINE(R)**Search Strategy:

| **#** | **Searches** | **Results** |
| --- | --- | --- |
| 1 | (((Care Group or Care Groups or Care Group Volunteers or Community Volunteer system or Community-based programme or Community-based or Community-Based Primary Health Care or CBPHC or Mother care groups or Social) and behavior change communication) or Primary care or Primary health care).af. | 178,221 |
| 2 | ((Maternal newborn and child health) or Nutrition or Undernutrition or Malnutrition or Health or Breastfeeding or Infant feeding or Maternal or Child or Under-5 or Infant or Newborn or MCH or MNCH or Child Survival or Child survival programme or Mortality).af. | 7,125,482 |
| 3 | ((Low and Middle Income Countries) or Resource-constrained or LMICs or Developing countries or Africa or Asia or (((((((AFGHANISTAN or ALBANIA or ALGERIA or AMERICAN SAMOA or ANGOLA or ARGENTINA or ARMENIA or AZERBAIJAN or BANGLADESH or BELARUS or BELIZE or BENIN or BHUTAN or BOLIVIA or BOSNIA HERZEGOVINA or BOTSWANA or BRAZIL or BULGARIA or BURKINA FASO or BURUNDI or CABO VERDE or CAMBODIA or CAMEROON or CENTRAL AFRICAN REPUBLIC or CHAD or CHINA or COLOMBIA or COMOROS or CONGO or COSTA RICA or COTE DIVOIRE or CUBA or DJIBOUTI or DOMINICA or DOMINICAN REPUBLIC or ECUADOR or EGYPT or EL SALVADOR or EQUATORIAL GUINEA or ERITREA or ESWATINI or ETHIOPIA or FIJI or GABON or GAMBIA or GEORGIA or GHANA or GRENADA or GUATEMALA or GUINEA or GUINEA BISSAU or GUYANA or HAITI or HONDURAS or INDIA or INDONESIA or IRAN or IRAQ or JAMAICA or JORDAN or KAZAKHSTAN or KENYA or KIRIBATI or KOREA or KOSOVO or KYRGYZ REPUBLIC or LAO or LEBANON or LESOTHO or LIBERIA or LIBYA or MADAGASCAR or MALAWI or MALAYSIA or MALDIVES or MALI or MARSHALL ISLANDS or MAURITANIA or MAURITIUS or MEXICO or MICRONESIA or MOLDOVA or MONGOLIA or MONTENEGRO or MOROCCO or MOZAMBIQUE or MYANMAR or NAMIBIA or NAURU or NEPAL or NICARAGUA or NIGER or NIGERIA or NORTH MACEDONIA or PAKISTAN or PAPUA NEW GUINEA or PARAGUAY or PERU or PHILIPPINES or ROMANIA or RUSSIAN FEDERATION or RWANDA SAMOA or SAO TOME) and PRINCIPE) or SENEGAL or SERBIA or SIERRA LEONE or SOLOMON ISLANDS or SOMALIA or SOUTH AFRICA or SOUTH SUDAN or SRI LANKA or ST LUCIA or ST VINCENT) and THE GRENADINES) or SUDAN or SURINAME or VSYRIAN ARAB REPUBLIC or TAJIKISTAN or TANZANIA or THAILAND or TIMOR LESTE or TOGO or TONGA or TUNISIA or TURKEY or TURKMENISTAN or TUVALU or UGANDA or UKRAINE or UZBEKISTAN or VANUATU or VENEZUELA or VIETNAM or WEST BANK) and GAZA) or YEMEN or ZAMBIA or ZIMBABWE)).af. | 373,024 |
| 4 | 1 and 2 and 3 | 10,460 |
| 5 | (Care Group or Care Groups or Care Group Volunteers or Community Volunteer system or Community-based programme or Community-based or Community-Based Primary Health Care).af. | 58,622 |
| 6 | 2 and 3 and 5 | 4,989 |
| 7 | (Nutrition or Undernutrition or Malnutrition or Health or Breastfeeding or Infant feeding).af. | 4,339,243 |
| 8 | 3 and 5 and 7 | 4,800 |
| 9 | ((Low and Middle Income Countries) or Resource-constrained or LMICs or Developing countries or Africa or Asia).af. | 360,383 |
| 10 | 5 and 7 and 9 | 4,645 |
| 11 | (Care Group or Care Groups or Care Group Volunteers or Community Volunteer system or Community-based programme or Community-based or Community-Based Primary Health Care).ab. | 47,979 |
| 12 | 7 and 9 and 11 | 3,986 |
| 13 | (Care Group or Care Groups or Care Group Volunteers or Community Volunteer system or Community-based programme or Community-based or Community-Based Primary Health Care).ti. | 13,410 |
| 14 | 7 and 9 and 13 | 1,172 |
| 15 | (Nutrition or Undernutrition or Malnutrition or Health or Breastfeeding or Infant feeding).ti. | 588,184 |
| 16 | 9 and 13 and 15 | 260 |

Database(s): **PsycInfo** Search Strategy:

| **#** | **Query** | **Limiters/Expanders** | **Last Run Via** | **Results** |
| --- | --- | --- | --- | --- |
| 1 | Care Group or Care Groups or Care Group Volunteers or Community Volunteer system or Community-based programme or Community-based or Community-Based  Primary Health Care or CBPHC or Mother care groups or Social and behavior change  communication or Primary care or Primary health care | Expanders  - Apply equivalent subjects Search modes  - Boolean/Phrase | Interface  - EBSCOhost Research Databases Search Screen  - Advanced Search Database  - APA PsycInfo | 104,501 |
| 2 | Maternal newborn and child health or Nutrition or Undernutrition or Malnutrition or Health or Breastfeeding or Infant feeding or Maternal or Child or Under-5 or Infant or Newborn or MCH or MNCH or Child Survival or Child survival programme or Mortality | Expanders  - Apply equivalent subjects Search modes  - Boolean/Phrase | Interface  - EBSCOhost Research Databases Search Screen  - Advanced Search Database  - APA PsycInfo | 2,079,883 |
| 3 | Low and Middle Income Countries or Resource constrained or LMICs or Developing countries or Africa or Asia or Expanders  - Apply equivalent subjects Search modes  - Boolean/Phrase or ANGOLA or Expanders  - Apply equivalent subjects Search modes  - Boolean/Phrase AFGHANISTAN or ALBANIA or ALGERIA or AMERICAN SAMOA or ARGENTINA or ARMENIA or AZERBAIJAN or BANGLADESH or BELARUS or BELIZE or BENIN or BHUTAN or BOLIVIA or BOSNIA HERZEGOVINA or BOTSWANA or BRAZIL or BULGARIA or BURKINA FASO or BURUNDI or CABO VERDE or CAMBODIA or CAMEROON or CENTRAL AFRICAN REPUBLIC or CHAD or CHINA or COLOMBIA or COMOROS or CONGO or COSTA RICA or COTE DIVOIRE or CUBA or DJIBOUTI or DOMINICA or DOMINICAN REPUBLIC or ECUADOR or EGYPT or EL SALVADOR or EQUATORIAL GUINEA or ERITREA or ESWATINI or ETHIOPIA or FIJI or GABON or GAMBIA or GEORGIA or GHANA or GRENADA or GUATEMALA or GUINEA or GUINEA BISSAU or GUYANA or HAITI or HONDURAS or INDIA or INDONESIA or IRAN or IRAQ or JAMAICA or JORDAN or KAZAKHSTAN or KENYA or KIRIBATI or KOREA or KOSOVO or KYRGYZ REPUBLIC or LAO or LEBANON or LESOTHO or LIBERIA or LIBYA or MADAGASCAR or MALAWI or MALAYSIA or MALDIVES or MALI or MARSHALL ISLANDS or MAURITANIA or MAURITIUS or MEXICO or MICRONESIA or MOLDOVA or MONGOLIA or MONTENEGRO or MOROCCO or MOZAMBIQUE or MYANMAR or NAMIBIA or NAURU or NEPAL or NICARAGUA or NIGER or NIGERIA or NORTH MACEDONIA or PAKISTAN or PAPUA NEW GUINEA or PARAGUAY or PERU or PHILIPPINES or ROMANIA or RUSSIAN FEDERATION or RWANDA SAMOA or SAO TOME and PRINCIPE or SENEGAL or SERBIA or SIERRA LEONE or SOLOMON ISLANDS or SOMALIA or SOUTH AFRICA or SOUTH SUDAN or SRI LANKA or ST LUCIA or ST VINCENT and THE GRENADINES or SUDAN or SURINAME or SYRIAN ARAB REPUBLIC or TAJIKISTAN or TANZANIA or THAILAND or TIMOR LESTE or TOGO or TONGA or TUNISIA or TURKEY or TURKMENISTAN or TUVALU or UGANDA or UKRAINE or  UZBEKISTAN or VANUATU or VENEZUELA or VIETNAM or WEST BANK and GAZA or YEMEN or ZAMBIA or ZIMBABWE | Expanders  - Apply equivalent subjects Search modes  - Boolean/Phrase | Interface  - EBSCOhost Research Databases Search Screen  - Advanced Search Database  - APA PsycInfo | 584,078 |
| 4 | S1 AND S2 AND S3 | Expanders  - Apply equivalent subjects Search modes  - Boolean/Phrase | Interface  - EBSCOhost Research Databases Search Screen  - Advanced Search Database  - APA PsycInfo | 12,994 |
| 5 | TI Care Group or Care Groups or Care Group Volunteers or Community Volunteer system or Community-based programme or Community-based or Community-Based Primary Health Care | Expanders  - Apply equivalent subjects Search modes  - Boolean/Phrase | Interface  - EBSCOhost Research Databases Search Screen  - Advanced Search Database  - APA PsycInfo | 8,355 |
| 6 | TI Nutrition or Undernutrition or Malnutrition or Health or Breastfeeding or Infant feeding | Expanders  - Apply equivalent subjects Search modes  - Boolean/Phrase | Interface  - EBSCOhost Research Databases Search Screen  - Advanced Search Database  - APA PsycInfo | 191,424 |
| 7 | Low and Middle Income Countries or Resource constrained or LMICs or Developing countries or Africa or Asia | Expanders  - Apply equivalent subjects Search modes  - Boolean/Phrase | Interface  - EBSCOhost Research Databases Search Screen  - Advanced Search Database  - APA PsycInfo | 71,803 |
| 8 | S5 AND S6 AND S7 | Expanders  - Apply equivalent subjects Search modes  - Boolean/Phrase | Interface  - EBSCOhost Research Databases Search Screen  - Advanced Search Database  - APA PsycInfo | 85 |
